# Modeling the spatial distribution of African buffalo (*Syncerus caffer*) in the Kruger National Park, South Africa

**DOI:** 10.1371/journal.pone.0182903

**Published:** 2017-09-13

**Authors:** Kristen Hughes, Geoffrey T. Fosgate, Christine M. Budke, Michael P. Ward, Ruth Kerry, Ben Ingram

**Affiliations:** 1 Department of Production Animal Studies, University of Pretoria, Onderstepoort, South Africa; 2 Veterinary Integrative Biosciences, Texas A&M University, College Station, Texas, United States of America; 3 Faculty of Veterinary Science, University of Sydney, Camden, Australia; 4 Department of Geography, Brigham Young University, Provo, Utah, United States of America; 5 Facultad de Ingeniería, Universidad de Talca, Curicó, Chile; Gaziosmanpasa Universitesi, TURKEY

## Abstract

The population density of wildlife reservoirs contributes to disease transmission risk for domestic animals. The objective of this study was to model the African buffalo distribution of the Kruger National Park. A secondary objective was to collect field data to evaluate models and determine environmental predictors of buffalo detection. Spatial distribution models were created using buffalo census information and archived data from previous research. Field data were collected during the dry (August 2012) and wet (January 2013) seasons using a random walk design. The fit of the prediction models were assessed descriptively and formally by calculating the root mean square error (rMSE) of deviations from field observations. Logistic regression was used to estimate the effects of environmental variables on the detection of buffalo herds and linear regression was used to identify predictors of larger herd sizes. A zero-inflated Poisson model produced distributions that were most consistent with expected buffalo behavior. Field data confirmed that environmental factors including season (P = 0.008), vegetation type (P = 0.002), and vegetation density (P = 0.010) were significant predictors of buffalo detection. Bachelor herds were more likely to be detected in dense vegetation (P = 0.005) and during the wet season (P = 0.022) compared to the larger mixed-sex herds. Static distribution models for African buffalo can produce biologically reasonable results but environmental factors have significant effects and therefore could be used to improve model performance. Accurate distribution models are critical for the evaluation of disease risk and to model disease transmission.

## Introduction

The population density of wildlife species that are reservoirs for disease has an important and complex relationship with the risk of disease in domestic hosts [1]. The rate of adequate contacts, the duration of infectiousness, herd immunity, and level of disease in the population are all important drivers of infectious disease transmission. Spatio-temporal distributions are frequently employed as approximations of animal contacts and therefore describing these distributions is an important first step in the development of disease transmission models. The hypothesis of the present study was that spatial heterogeneity in African buffalo numbers could be explained by herd-level and environmental predictors.

Herd behavior, critical population threshold, and disease transmission parameters are important considerations for modeling etiologies that have wildlife reservoirs [1]. Disease transmission occurs through the direct or indirect contact of an infected host with a susceptible animal [2]. Many modeling approaches assume that contacts occur via a homogenous mixing population, but more complex contact patterns are better approximations to reality [3]. Infectious disease models should therefore account for herd-level factors to be more accurate representations of disease transmission.

The African buffalo (*Syncerus caffer*) is a large, non-domestic bovid found throughout much of southern Africa. Distribution patterns are influenced by season, topography, and herd size [4]. The animals must drink at least once per day and this requirement controls daily movements, especially during the dry season [5]. Buffalo herds vary tremendously in size [6, 7]. Herds range from one individual to over 1000 and the herd sizes in a population tend to follow an exponential distribution or power curves with negative exponents [6]. Herds consist mainly of related females and their offspring, with males joining during the breeding season. Older males are often solitary or form small bachelor herds.

Kruger National Park (KNP), situated within the northeastern region of South Africa, is one of Africa’s largest wildlife reserves. Foot-and-mouth disease (FMD) is endemic in KNP and African buffalo are the reservoir host for the three Southern African Territories serotypes (SAT 1–3) of the FMD virus [8, 9]. African buffalo are believed to be the major source of FMD virus transmission to domestic livestock in the areas surrounding KNP [10]. Stray buffaloes pose a risk [11] and gaps in game-proof fences can be used by wildlife to escape, or livestock to enter [12, 13]. Transmission events frequently occur due to close contact between acutely infected and susceptible animals [14]. The spatial distribution of buffalo in KNP is therefore expected to affect the risk of FMD virus transmission to livestock in surrounding areas.

There are few published models related to the spatial distribution of African buffalo. Regression techniques including spatial autocorrelation have been used to predict African buffalo occupancy in KNP [15], but distribution maps were not presented in the published report. African buffalo movement patterns have also been modeled [16], but individual movement patterns were not generalized to population-level distributions. Forage quality and water availability are known to influence habitat usage [17–21] but occupancy or distribution maps have not be published based on these data.

Animal distribution models are important for disease transmission modeling and risk assessment. Disaggregation is a common technique used to create distribution maps from aggregated livestock data [22]. Regression techniques incorporating spatial autocorrelation [15] and Poisson kriging have both been used previously to describe the distribution of wildlife in KNP [23]. No previous studies could be identified that compared modeling strategies nor formally assessed the impact of including spatial autocorrelation on the validity of produced animal distributions. The aim of this study was to model the African buffalo (*Syncerus caffer*) distribution of KNP using four different modeling approaches. We hypothesized that there would be spatial heterogeneity in the buffalo distribution that could be partially explained by environmental factors.

## Materials and methods

### Study site

**Fig 1:**
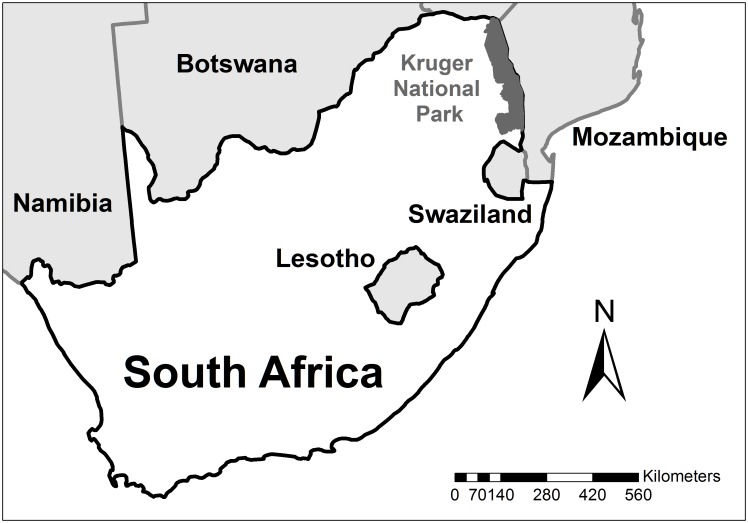
Kruger National Park’s location within South Africa and other southern African countries.

The study site was the Kruger National Park (KNP), approximately 20,000 km^2^ in the northeastern region of South Africa (Fig 1). It has a subtropical climate with a wet season (October to March) and a dry season (April to September). Rainfall only typically occurs during the wet season and the amount of rain also varies spatially. The park has an annual average of 750 mm of rain in the south compared to 440 mm in the north. The vegetation also differs greatly within the park with a large amount of thick mopane shrubland in the north and open grassland savanna in the south [24].

### Data collection

The study included environmental data produced for the study area, buffalo census data from the study area, tracking data collected from collared buffalo within smaller regions of the study area, and field data collected for model evaluation. All data used for the development of the animal distribution models were obtained from the South African National Parks (SANParks) repository. Ground cover variables (herbaceous cover, tree cover, bare ground) were originally derived from Moderate Resolution Imaging Spectroradiometer (MODIS) data [25]. Environmental data included river locations, soil composition, mean annual rainfall, and landscape types. Buffalo census data, which are collected by helicopter-based surveys during the dry season, were obtained for the 11 years preceding the study (2001–2011). During the census, helicopters fly along the major rivers driving the buffalo into smaller areas to facilitate counting. Tracking data from collared buffalo were collected during previous research projects [26, 27]. The current study was approved by the Animal Ethics Committee of the Faculty of Veterinary Science, University of Pretoria (Project No. V083-11).

**Fig 2:**
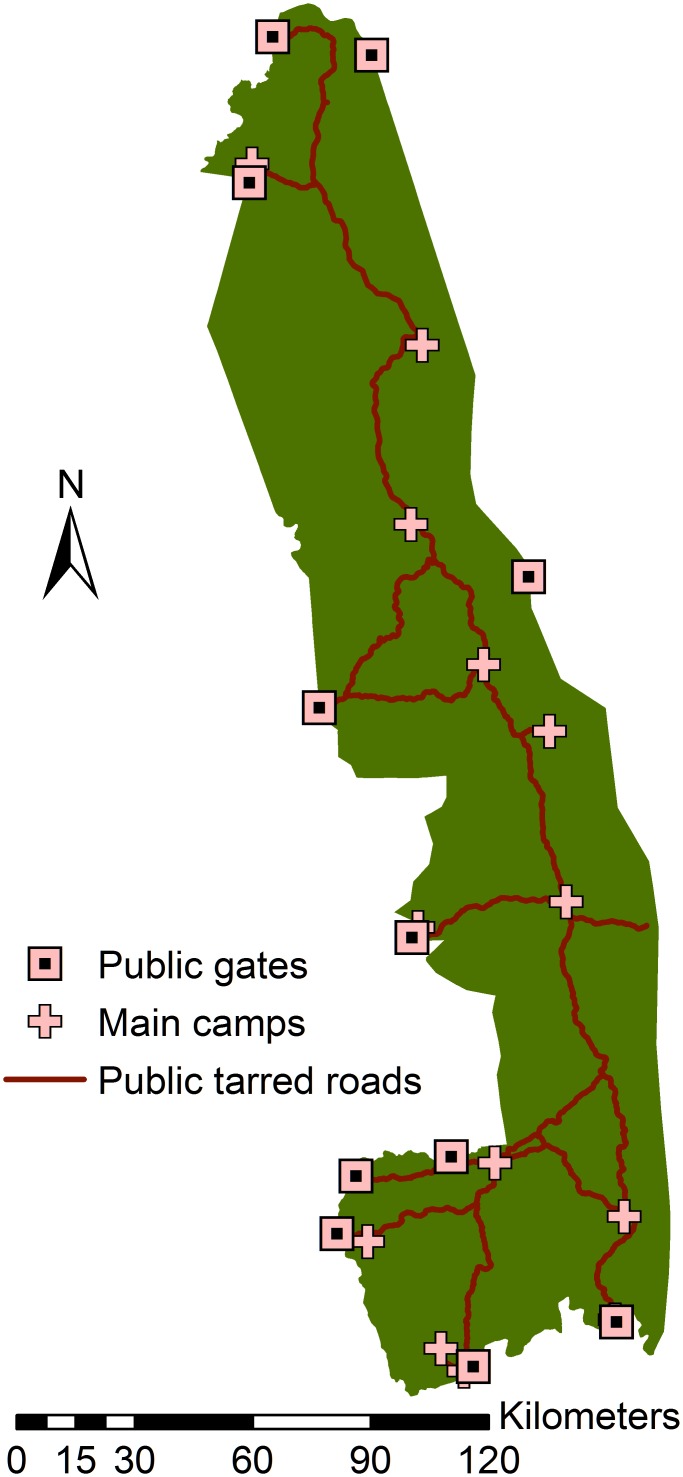
Kruger National Park public gates, camps available for overnight stays, and tarred public roads.

The KNP road network was chosen as the method to collect field data due to feasibility and costs associated with other sampling approaches. The public road network of KNP is irregular and therefore a random walk [28] design was used to collect field data rather than the more common quadrant or transect sampling approaches. Random route approaches are more commonly used in sociological studies despite the potential for selection bias [29]. One data set was collected during the dry season (August 2012) and another during the wet season (January 2013). The KNP was divided into three areas (northern, central, and southern) for field data collection. Four locations were purposely selected within each KNP area that allowed for the most divergent starting locations (Fig 2). The starting area for each sampling trip (August and January) was randomly selected and then the order of daily starting locations was also determined randomly. It was necessary to stratify KNP into three areas for sampling to reduce the required distance to get to the next starting location after the completion of the daily sampling. A route was created from each starting location: a random number generator was used to determine turning direction (0-back, 1-forward, 2-left, 3-right) based on a map of the KNP public road network. Each starting location was assigned a numerical value and a random number generator was again used to determine the sequence of starting locations. Each starting location was used twice, thus producing a total of 24 routes. Each observation day consisted of a five-hour drive along the random route, which was determined prior to the sampling day using GIS maps. Data were collected when buffalo were observed within 250 m of the observation vehicle. Distance to the spotting vehicle was determined using a golf spotting scope (Binolux Golf Scope, Compass Industries, Inc., NY, USA) and buffalo numbers were manually counted. Additional data that were collected included the GPS location of the buffalo, herd composition (bachelor or mixed), time of day, temperature, humidity, barometric pressure, visible water source, savanna type, and a subjective measure of vegetation density. A dense landscape was defined as an area that contained trees or shrubs covering 50% or more of the area adjacent to the road and in which the buffalo were situated. An open landscape was one in which 5% or less of the area of interest contained trees or shrubs. Data were also recorded at the start of daily sampling and at the end of each hour during sampling. Data collection during the January sampling was not completed due to flooding of KNP and the subsequent closing of public roads.

### Spatial distribution modeling

#### Disaggregation based on environmental variables

The disaggregation model was based on an environmental dataset that consisted of mean annual rainfall, ground water distribution, soil type, savanna type, rivers, percent herbaceous cover [30], percent tree cover, and percent bare ground. All data were imported into commercial software (ArcGIS Desktop 10.3 ESRI Corp. Redlands, CA, USA) for spatial analysis and the base spatial unit was a 1 km^2^ grid cell. Buffers were applied to the rivers shapefile at an arbitrarily chosen 5-km interval (5, 10…30) using the Buffer function within the Analysis Tools of the ArcToolbox. This was done to determine the distance of buffalo to the nearest water source. A previously developed landscape classification system was used to create two predictor variables for savanna and soil types [31]. One kilometer square grids were created using the Fishnet function within the Feature Class component of the Data Management Tools of the ArcToolbox. Each fishnet grid cell was assigned a savanna type and a soil type based on the category that covered the majority of the area and was performed using the Zonal Statistics function within the Zonal component of the Spatial Analyst Tools of the ArcToolbox. Layers were joined into a single data set for each 1 km^2^ cell. The resulting dataset was a matrix of 19,575 grid cells classified by nine environmental characteristics: annual rainfall, ground water distribution, percent herbaceous cover, percent woody cover, percent bare ground, proximity to a water source, major versus minor water source, soil type, and savannah type.

Buffalo census data were assigned to the same fishnet grid cell system used to summarize the environmental data and averaged for the eleven-year study period (2001–2011). The exception was the data corresponding to the proximity to water source variable. Tracking data were used to calculate the suitability index for this variable because census data are biased; during the census buffalo are counted along the major rivers. The suitability analysis was performed independently for each of the nine environmental predictor variables. The total number of buffalo observed in each cell type was divided by the total number of grid cells of that same type. An overall suitability index for each grid square was calculated as the sum of the indexes for all predictor variables. The approach yields an equivalent result as calculating an unweighted mean of the indexes. The equal weighting of predictors has been used previously for a different wildlife species [32] and a valid system of weighting for African buffalo was not available from the literature. The 2011 population total (42,163 buffalo) was distributed across the KNP proportional to calculated suitability values to produce estimated locations for the 2011 population.

#### Poisson kriging

A previously described method of Poisson kriging used for transect lines [23] was modified to account for irregularly spaced census information. Thiessen polygons, the area around a point that is closest to that point than any other point in the dataset, were created around the locations of observed buffalo in the census data. These polygons were formed by the perpendicular bisectors of the line segments between each pair of points. Spatial densities were calculated as the count of buffalo in each polygon divided by its corresponding area. SpaceSTAT (BioMedware, Ann Arbor, MI, USA) was used to compute Poisson variograms and to Poisson krige from the census data to the August 2012 and January 2013 field data validation points. Census data were then Poisson kriged to the 1 km^2^ grids to create a continuous surface. The detection probability of smaller groups of buffalo is assumed to be less than a larger herd occupying the same spatial area. For this reason, counts associated with low spatial densities (ie lower numbers of buffalo in larger areas) are less likely to be accurate and were therefore down-weighted during the kriging process. The kriging function was weighted to give more importance to more reliable data pairs based on small observational areas [33, 34]. One divided by the observational area was used as the denominator for each observation as the weighting factor for this reason. A complete description of the Poisson kriging approach can be found in the previous publications [33, 34].

#### Zero-inflated Poisson (ZIP) model

A zero-inflated Poisson (ZIP) model is the combination of logistic and Poisson regression likelihood functions [35]. The log likelihood is the multiplication of the binary component and a Poisson model truncated at zero. Zero-inflated Poisson models were built using the 1 km^2^ grid environmental data described previously. However, quantitative variables were first standardized so that each distribution had a mean value of zero and a standard deviation of one. The number of buffalo from the tracking data were summed using the Zonal Statistics function within the Zonal component of the Spatial Analyst Tools of the ArcToolbox. Calculations were performed based on the same fishnet used to summarize environmental predictors. Environmental data were linked to the tracking data counts and exported for analysis within commercial statistical software (STATA version 11.0, StatCorp, College Station, TX, USA). The statistical outcome for the analysis was the buffalo counts and the environmental variables were evaluated as predictors. Environmental variables were first assessed for collinearity using Spearman’s rho and only a single variable was retained if the correlation coefficient was > 0.9. A high criterion was chosen due to the large data set and the expectation that environmental predictor variables would be highly correlated. A backwards step-wise approach was used to build a reduced main effects model. Environmental predictors were removed one-by-one based on the largest Wald P value in either the logistic or Poisson component of the likelihood function. Each removed variable was subsequently added back into the reduced model one-by-one and retained if inclusion improved the McFadden's adjusted pseudo-r^2^ value. Regression coefficients from the final main effects only model were inserted into program code (WinBugs version 1.4, MRC Biostatistics Unit) to fit a ZIP model using standardized environmental predictor data for the entirety of the KNP. Predicted buffalo counts per 1 km^2^ grid cell were adjusted to a total population of 42,163 individual buffalos (2011 census).

#### Conditional autoregressive (CAR)

A zero-inflated Poisson (ZIP) model incorporating spatial dependence terms was developed through the modification of a previously published technique [36]. The general model form follows that of the previous (ZIP) section but also includes a bivariate conditional autoregressive (CAR) random effect to account for spatial autocorrelation [37].

Markov chain Monte Carlo (MCMC) techniques using available statistical software (WinBugs version 1.4, MRC Biostatistics Unit, Cambridge, UK) were used to evaluate multiple CAR models. A spatial matrix for the entire park was created using available software (GeoDa Center for Geospatial Analysis and Computation, The University of Chicago, Chicago, Illinois, USA). The matrix was developed using rook weighting and converted to WinBugs code using the web-based exploratory spatial data analysis tool available at GeoDa (http://spatial.uchicago.edu/geoda-web). The CAR models were implemented with a 100,000 iteration burn-in that only retained every fifth iteration to reduce autocorrelation. Predicted values were obtained from an additional 20,000 iterations after burn-in. Convergence was assessed by evaluating plots of parameter iterates and by calculating the Gelman-Rubin statistic. Predicted buffalo counts per 1 km^2^ grid cell were then adjusted to the total population of the 2011 buffalo census.

A base CAR model was developed that only included spatial autocorrelation; no environmental predictors were included in either the logistic or Poisson component of the ZIP model (S1 Code). Subsequent CAR models contained the spatial autocorrelation terms in addition to environmental variables. Standardized environmental data and buffalo counts from the tracking data were used in the CAR models. Multiple models were evaluated, including limiting continuous variables to the Poisson equation and categorical predictors to the logistic component. A backwards stepwise model building approach was performed in effort to identify an adequate prediction model. Convergence was evaluated as described previously.

### Data analysis

Field data from the road-based survey were spatially projected using commercial software (ArcGIS Desktop 10.3 ESRI Corp. Redlands, CA, USA). The 1 km^2^ fishnet developed for the spatial prediction models was used to extract data from the field observations using the Join function within the GIS software. Predictions from all four modeling methods were linked in this manner. The KNP road network was used to make field observations and therefore only the 1 km^2^ grid cells adjacent to the travelled roads were used for evaluation of spatial models. The shapefile of the travelled roads (3,216 1 km^2^) was spatially joined to the fishnet including the model predictions and observed buffalo counts. The resulting attribute table was exported for analysis in a commercial spreadsheet program (Microsoft Office Excel 2010, Microsoft Corporation, Redmond, WA). A subset of the linked data corresponding to the central portion of the park (from the line marking the Tropic of Capricorn south to the level of the Talamati camp; latitude: -23.5 to -24.6; 1,355 1 km^2^ grid cells) was selected using the select by polygon tool in the GIS software and exported. This restriction was performed due to flooding in KNP during January 2013, which prevented complete data collection for the wet season.

The model evaluation data were analyzed by entering formulas into the spreadsheet program. The sum of squared errors (SSE) was calculated as the sum of the squared deviations between the observed and predicted buffalo counts for each 1 km^2^ grid cell after transformation using the natural logarithm. This sum was divided by the total number of observations and the square root was taken to calculate the root mean square error (rMSE), which was used to descriptively compare the fit of the four models. Lower values of the rMSE indicate better predictive ability of the model. Spearman’s rho was also used to compare model predictions to collected road-based observations.

Herd size data collected during the road-based survey were assessed for normality by plotting histograms, calculating descriptive statistics, and performing the Shapiro-Wilk test. Data violated the normality assumption and were transformed using the natural logarithm to improve the distributional form prior to statistical analysis. Correlations between quantitative data were estimated using Spearman’s rho. The associations between detection of a buffalo herd and environmental predictors were estimated using logistic regression. Locations where buffalo were observed were compared to the hourly environmental data collection sites (no buffalo). Logistic regression was also used to identify environmental predictors of bachelor buffalo versus mixed-sex herds. Linear regression was used to identify significant predictors of increasing herd size. In all situations, univariate screening models were fit and all variables where P < 0.20 were included for building multivariable models. Multivariable models were built using a backwards stepwise procedure and each variable was removed one-by-one based on the largest Wald (logistic regression) or F test (linear regression) P value. Three-level categorical variables were reclassified as dichotomous when one of the levels had a significant Wald statistic in absence of overall variable significance. The stepwise procedure continued until all remaining main effect terms were P < 0.05. Each variable removed during the stepwise procedure was individually entered back into the reduced model and retained if the factor was significantly associated with the outcome. Models were constructed to investigate the primary effects of variables and therefore interaction terms were not assessed. The fit of the final multivariable models were assessed based on Hosmer-Lemeshow goodness-of-fit tests (logistic regression) or r^2^ calculations (linear regression). Statistical analyses were performed in commercially available software (IBM SPSS Statistics 23, International Business Machines Corp, Armonk, NY, USA) and results were interpreted at the 5% level of significance.

## Results

The disaggregation model produced predicted values for herd sizes ranging from zero to five (interquartile range (IQR) 2–2), with the majority of cells containing two buffalo. Predictions from the Poisson kriging model suggested that herds typically contained less than ten animals (IQR 0–3). There were scattered locations that contained large herd predictions (approximately 1% of the total area); however, most areas contained predictions of less than 10 buffalo (the majority of cells were predicted to have zero buffalo). The ZIP model produced the most biologically reasonable distribution map based on herd sizes ranging from 1 individual to over 500 and the spatial distribution was less uniform than the other modeling approaches. The majority of cells contained no buffalo, but almost half of the area was predicted to contain herds that ranged from less than ten animals to over 200 animals (IQR 0–1). A CAR model including only spatial correlation (no environmental data) produced predictions with reasonable values (range 0–222, IQR 0–1) but an unrealistic distribution clustered around the location of the data used for development of the model (tracking data). The four model prediction maps are presented as supplemental material (S1 Fig). CAR models that included environmental predictor variables (ZIP model including the spatial autocorrelation) would not converge and, therefore, no distribution maps could be produced.

All models had similar rMSE values with limited variability between the two sampling seasons (Table 1). The CAR model, which did not contain any of the environmental variables, performed the best out of the four models when evaluated for the entire KNP despite the unrealistic spatial distribution that was produced. The ZIP model performed the best based on the central-only subset of KNP. This model was the second best (based on relative rMSE) when evaluated for the entire KNP. This model also predicted populations that were dispersed throughout the park rather than the clustered values predicted by the CAR model. However, the ZIP model did not produce predictions that were significantly correlated with the collected field data.

**Table 1:** Root mean square error (rMSE) and Spearman’s rho correlation between predicted and observed buffalo counts for observations in Kruger National Park (KNP), South Africa during 2012. Data presented for the entire KNP road network and a subset of the central portion of the park corresponding to the region with complete sampling during both seasons.

Region	Model	Date	rMSE	Spearman’s rho (P value)
Entire KNP	Zero-inflated Poisson	August 2012	2.17	0.027 (0.121)
		January 2013	2.10	-0.017 (0.347)
	Poisson kriging	August 2012	2.44	-0.033 (0.064)
		January 2013	2.35	0.052 (0.003)
	Conditional autoregressive	August 2012	1.79	0.077 (<0.001)
		January 2013	1.71	0.027 (0.132)
	Disaggregation	August 2012	2.61	-0.014 (0.440)
		January 2013	2.56	-0.022 (0.214)
Central area	Zero-inflated Poisson	August 2012	2.18	0.051 (0.061)
		January 2013	2.02	-0.053 (0.053)
	Poisson kriging	August 2012	2.50	0.009 (0.751)
		January 2013	2.39	0.058 (0.034)
	Conditional autoregressive	August 2012	2.37	-0.021 (0.435)
		January 2013	2.24	-0.020 (0.467)
	Disaggregation	August 2012	2.59	-0.006 (0.831)
		January 2013	2.50	0.015 (0.591)

**Fig 3:**
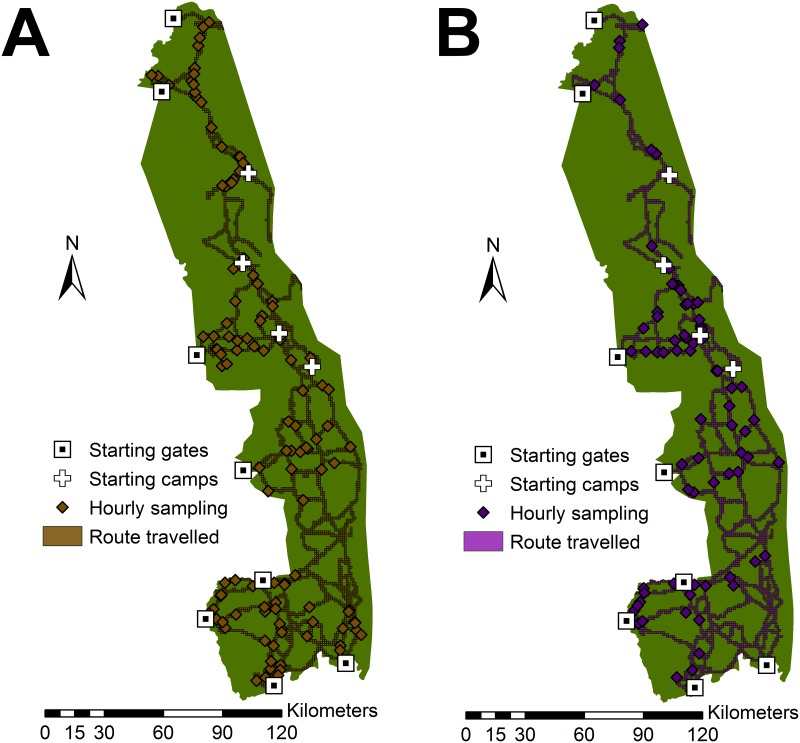
Zero-inflated Poisson regression model predictions of buffalo herd sizes within Kruger National Park overlaid with observed herds during the dry season (August 2012, left pane) and the wet season (January 2013, right pane).

The results from the ZIP model were the most consistent with buffalo behavior and despite the lack of a significant correlation, the observations were relatively consistent with the predicted high density areas (Fig 3). The subjective consistency of the observations appeared stronger for the sampling that was performed during the dry season. However, there was an apparent lack of consistency in the southern region of KNP where very few herds were observed despite areas of large predicted herd sizes.

**Fig 4:**
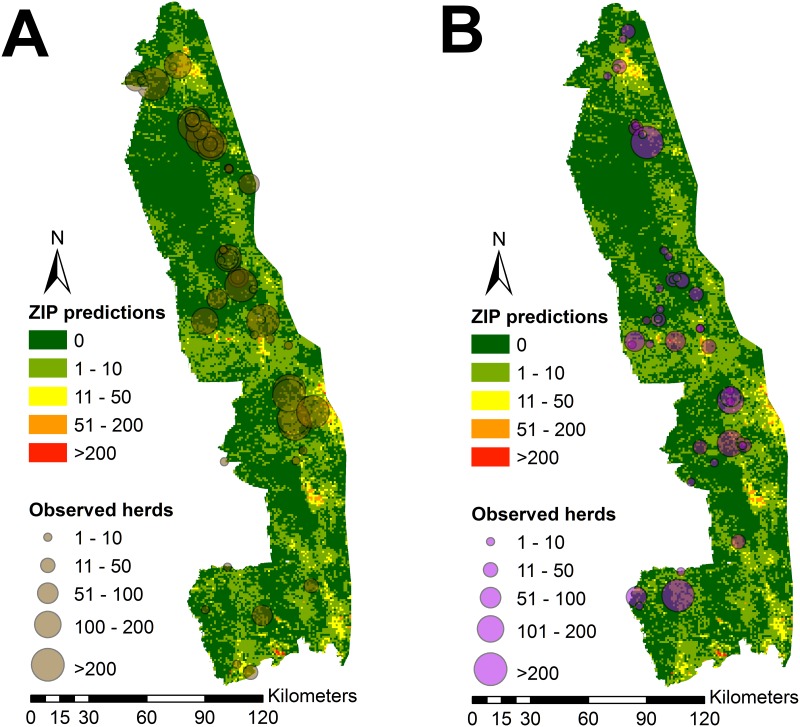
Field sampling starting locations and routes travelled during the dry season field observations (August 2012, left pane) and the wet season (January 2013, right pane).

The roads travelled for field data collection were the same during the dry and wet seasons (Fig 4), but only 57% of the sampling (16/28 days) was completed during the wet season (January 2013) because of heavy rains and flooding of the road network. The median observed herd size during the dry season was 45 animals (range 1–1,200; IQR 3–100; n = 37) and 4.5 animals (range 1–250; IQR 2–46; n = 34) during the wet season. More buffalo groups were observed during the wet season (P = 0.008), in bush-type vegetation (P = 0.002), in lower vegetation density areas (P = 0.010), and in northern regions of KNP (P < 0.001; Table 2; S1 & S2 Tables). Bachelor buffalo herds were more frequently observed during the wet season (P = 0.022) and in denser vegetation (P = 0.005) compared to the mixed-sex herds (Table 3; S3 Table). Bachelor herd sizes were smaller than the mixed-sex herds (P < 0.001; Table 4; S4 Table). When adjusting for bachelor herds, dry season herds (P = 0.029), herds observed in bush-type vegetation (P = 0.019), and herds observed in less dense vegetation (P < 0.001) were significantly larger. Collected field data are available as Supporting Information (S1 File).

**Table 2:** Multivariable logistic regression to identify predictors of observing buffalo herds based on field data collected for 105 detected herds of buffalo in Kruger National Park during August 2012 and January 2013 compared to 234 hourly time points without buffalo observations.

Variable	level	Buffalo observations (n)	Total locations (n)	Odds ratio (95% CI)	Wald P value
Season	Dry (August)	56	200	0.50 (0.30, 0.83)	0.008
	Wet (January)	49	139	Referent	
Vegetation type	Bush	78	203	2.39 (1.37, 4.15)	0.002
	Mixed or tree	27	136	Referent	
Vegetation density	More open	26	54	2.36 (1.22, 4.54)	0.010
	Other	79	285	Referent	
Latitude	Northern region	54	115	3.83 (2.26, 6.47)	<0.001
	Other	51	224	Referent	

CI = confidence interval.

Hosmer and Lemeshow chi-square = 2.89, df = 6, P = 0.823.

**Table 3:** Multivariable logistic regression to identify predictors of observing bachelor herds in 104 herds* of buffalo in Kruger National Park identified during August 2012 and January 2013.

Variable	level	Buffalo observations (n)	Total locations (n)	Odds ratio (95% CI)	Wald P value
Season	Dry (August)	21	55	0.38 (0.16, 0.87)	0.022
	Wet (January)	29	49	Referent	
Vegetation density	Dense	18	25	4.24 (1.53, 11.7)	0.005
	Other	32	79	Referent	

*The herd type could not be determined for one herd.

CI = confidence interval.

Hosmer and Lemeshow chi-square = 1.019, df = 2, P = 0.601.

**Table 4:** Multivariable linear regression for the estimation of effects of predictor variables on observed buffalo herd size* in 104 herds† of buffalo in Kruger National Park identified during August 2012 and January 2013.

Variable	level	Total herds (n)	Slope estimate (95% CI)	Student’s t P value
Bachelor herd				
	No	50	3.10 (2.73, 3.46)	<0.001
	Yes	54	Referent	
Season				
	Dry (August)	56	0.40 (0.04, 0.76)	0.029
	Wet (January)	49	Referent	
Vegetation type				
	Bush	78	0.49 (0.08, 0.90)	0.019
	Mixed or tree	27	Referent	
Vegetation density				<0.001
	More open	26	0.86 (0.34, 1.38)	0.001
	Middle density	54	0.44 (0.01, 0.88)	0.047
	More dense	25	Referent	

*Analysis performed on the natural logarithm transformed herd size.

^†^Herd type could not be determined for one herd.

CI = confidence interval.

r^2^ = 0.805, adjusted r^2^ = 0.795.

## Discussion

The study reported here estimated the spatial distribution of the African buffalo (*Syncerus caffer*) of KNP using four different modeling approaches to assess the suitability of approaches for developing disease transmission models. African buffalo herds vary greatly in size, from 1 individual to more than 1000, and the probability distribution of herd sizes in a population tends to follow exponential distributions or negative power curves [6]. A biologically reasonable herd size distribution is one in which both very small and very large herds are present with an average of approximately 250 individuals [7]. The hypothesis of the present study was that spatial heterogeneity in African buffalo numbers could be explained by herd-level and environmental predictors. We also evaluated whether or not spatial autocorrelation would be important because regression approaches that exclude spatial autocorrelation over-estimate the effects of landscape variables [38]. The results of the present study suggest that the presence of African buffalo depends upon the herd structure and environmental factors but the effects of spatial autocorrelation were inconclusive.

There was no apparent seasonal effect on prediction error or correlation when the four modeling approaches were evaluated. The disaggregation method provided results that distributed the population too evenly over the entire park causing low population values in every cell. These are unrealistic predictions based on the known herd dynamics of the African buffalo. This method was developed for the modeling of white-tailed deer populations in Texas [32], which have different behaviors and herd dynamics. White-tailed deer are often found in much smaller groups; therefore, this broad spatial dispersal would be a more realistic expectation. In the present study involving African buffalo, the main challenge was to account for the large variation in herd sizes. Further work on developing suitability values may produce a more realistic outcome from the disaggregation method. The calculation method utilized in the current study also did not allow for any environmental factor to have a negative effect and therefore each cell was required to have a positive value.

The CAR model that contained only the spatial autocorrelation term produced a distribution with realistic herd sizes but only within small sections of KNP. This was likely due to the data that were used to develop the model: GPS tracking data from previous studies in only the central and northern regions of KNP. A CAR model containing both the spatial autocorrelation term and environmental variables might produce more accurate maps. However, this model requires additional work. The inclusion of the environmental variables created a model that was presumably too complex and results were unstable as indicated by the lack of convergence.

The Poisson kriging model produced a biologically reasonable distribution despite not including environmental variables. The inclusion of such terms in a Poisson kriging model may further improve predictions. The theory underlying spatial autoregressive models, including kriging, is that cells closer to each other will be more similar than cells further apart (the First Law of Geography). This idea conflicts (at this 1 km^2^ cell size) with what is known of buffalo populations. An area containing a large herd is actually unlikely to be neighbored by another large herd even if the cells have similar suitability values. Therefore, this method may be a better representation of buffalo land preference rather than a true distribution. The rMSE values for the Poisson kriging model were slightly less than that of the disaggregation model.

The ZIP model created in this study produced a biologically reasonable distribution with the lowest rMSE for the area of KNP with complete sampling. This suggests that environmental variables are likely better predictors of buffalo presence rather than spatial autocorrelation. Field data collection confirmed the importance of environmental factors for the detection of buffalo herds. Vegetation type, vegetation density, and region within KNP were all strong predictors of buffalo herd detection. Predictors also varied by herd type (bachelor versus mixed-sex herds) suggesting that more accurate models could be produced by independently modeling the distribution of these two herd types. Herd size also varied by season and this effect was independent of the concurrent effect of bachelor herds. Field observations were reasonably consistent with ZIP model predictions, but the accuracy of the model would likely be improved by accounting for season and type of herd in the modeling procedure. The creation of multiple maps based on time of year and herd type would be expected to improve model predictions.

The collected field data (road-based buffalo counts) in the central and northern parts of KNP were reasonably consistent with ZIP model predictions. However, the southern area of KNP was also predicted to have areas that would be suitable for large buffalo herds, but field observations were inconsistent with these predictions. The southern part of KNP has a more extensive road network and it is unclear why relatively few buffalo were observed in this area. It is possible that the buffalo numbers are lower than expected or simply that the detection probability is different in this region of KNP for an unknown reason. The current study is not able to provide an explanation for an actual lower number of buffalo, but this could be due to possible environmental changes that occurred subsequent to the creation of the data sets employed in the study. It is further possible that a real reduction in buffalo numbers could occur due to disease or human-related factors. Bovine tuberculosis could be a possible cause of a population reduction as it was first recognized in the southern region of KNP before spreading northward [39].

There was little difference in the statistical fit of prediction models between seasons. Each model performed only marginally better in the wet season compared to the dry season evaluation data. The limited seasonal variability was unexpected. This observation was not consistent with our expectation that there would be a greater variation between seasons due to changes in buffalo behavior between the dry and wet seasons. Furthermore, it was expected that predictions would be more consistent with the dry season observations since the dry season is when the buffalo census is performed.

Two different buffalo data sets were used for the development of prediction models. The suitability values in the disaggregation method were developed using data from previous buffalo census surveys. The Poisson kriging model was also based on these census data. However, the ZIP and CAR models were based on tracking data collected from two previous studies. Tracking data were considered a better representation of buffalo land use, but only covered small portions of the park and, therefore, did not contain adequate data coverage for all environmental variables. The different data sources were necessary to cover the entire KNP, but this is a potential confounder for the comparison of models. Furthermore, different data sets were collected during different time periods and this was another potential source of bias in estimated distribution maps and the comparison of modeling approaches.

Observational bias might have occurred due to errors in detection that might have varied over time or locations in KNP. It is possible that herds were more likely to be observed early in the observation periods and fatigue decreased detection probability as sampling progressed. The random starting points and routes were employed in effort to reduce the impact of this time effect. However, the random walk approach using only the public road network might have also introduced errors due to variable coverage of KNP. The total distance travelled in each area of KNP was not recorded on a daily basis and this prevented a formal comparison of the observation time spent in the different regions. The density of hourly environmental sampling points suggested relatively good coverage of the entire public road network despite the lack of a formal comparison. A systematic approach such as quadrant or transect sampling would have ensured a more uniform sampling coverage. The potential effect of time varying factors such as fatigue were subjectively considered more important during study design, but it is unknown if this approach introduced more sampling error than it prevented. Using airplanes to fly transects [40] and the use of 4x4 vehicles with game rangers to sample randomly selected locations were not feasible data collection options within the context of this study. The KNP is a common tourist destination and wildlife do not appear to shy away from roads or people observing from cars. However, a formal evaluation of a potential road bias has not been published and the current study merely assumed that such a bias does not exist.

It would be beneficial for future work to use Poisson kriging to account for observational biases in a temporal analysis of counts from different census combined with tracking data. Such analysis could produce probability maps of finding a herd of a given size in a particular 1 km^2^ grid cell by analyzing the proportion of times various cells had certain herd sizes and analyzing the spatial autocorrelation in these proportions. As noted in the methods, expecting positive spatial autocorrelation in raw buffalo herd numbers is counter-intuitive. Large herds of buffalo tend to be found surrounded by large areas of few if any buffalo. This is negative spatial autocorrelation. The Local Moran’s I statistic could also be used to identify significant spatial outliers exhibiting this negative spatial autocorrelation. It would also be possible to devise a Poisson kriging approach that incorporated environmental data into the estimation process.

Visibility was impaired in some areas during road-based field data collection due to higher vegetation density. Only cells adjacent to the road network were included for the calculation of rMSE to decrease the effect of this bias. A possible source of confounding might have been changes in weather conditions because African buffalo prefer different habitats depending on the predominant weather pattern. The prediction error was evaluated for each model in both seasons to determine if this was a significant source of bias in the study design; however, little difference was noted between seasons. A further limitation is that the use of different datasets during the development of the models prevented the direct comparison of model validity using random subsets of the data for model development and validation. The objective of this study was to evaluate models using direct observations in the field but flooding during data collection and limited observations of buffalo reduced the ability to detect significant differences. Full data sets for both dry and wet seasons might have demonstrated more variability in model accuracy between seasons.

### Conclusion

Collected field data were not strongly correlated with any of the evaluated models but the zero-inflated Poisson model produced the most biologically plausible distribution map of African buffalo in the KNP, followed by the Poisson kriging model. These static distribution maps were reasonably consistent with observed animal densities. However, new approaches are necessary if these models are to be used for FMD risk assessments and developing disease transmission models. Future research should investigate the use of CAR models capable of adjusting for seasonal variations in herd structures in addition to spatial dependencies.

## Supporting information

S1 Fig a) Disaggregation model predictions (upper left); b) Poisson kriging model predictions (upper right); c) Zero-inflated Poisson model predictions (lower left); d) Conditional autoregression model predictions (lower right).
(TIF)

S1 Table The comparison of quantitative variables between locations where buffalo were observed in Kruger National Park during August 2012 and January 2013 compared to hourly (non-buffalo) observations and the correlation of these variables with the observed herd sizes. (DOCX)

S2 Table Univariate logistic regression to identify predictors of observing buffalo herds based on field data collected for 105 detected herds of buffalo in Kruger National Park during August 2012 and January 2013 compared to 234 hourly time points without buffalo observations. (DOCX)

S3 Table Univariate logistic regression to identify predictors of observing bachelor herds in 104 herds of buffalo in Kruger National Park identified during August 2012 and January 2013. (DOCX)

S4 Table Univariate linear regression for the estimation of effects of predictor variables on observed buffalo herd size in 104 herds of buffalo in Kruger National Park identified during August 2012 and January 2013. (DOCX)

S1 Code WinBUGS code for performing the conditional autoregressive (CAR) model. (DOCX)

S1 File Collected field data saved as comma separated file. (CSV)
