# Supplementary figures and images for: Modeling the spatial distribution of African buffalo (Syncerus caffer) in the Kruger National Park, South Africa
Source: PLoS One. 2017 Sep 13;12(9):e0182903. doi: 10.1371/journal.pone.0182903 (PMC5597095; doi:10.1371/journal.pone.0182903)

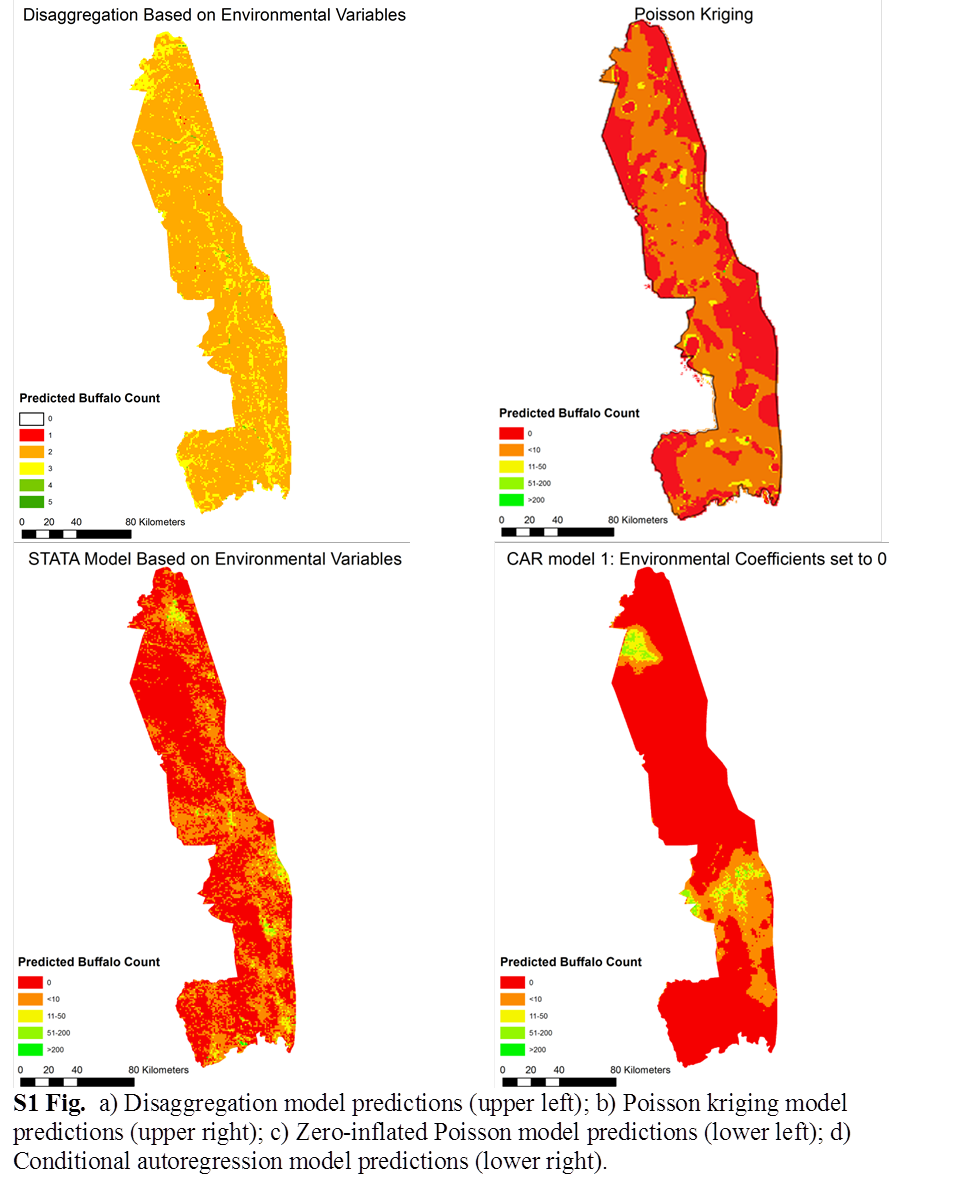

Supplement: S1 Fig — a) Disaggregation model predictions (upper left); b) Poisson kriging model predictions (upper right); c) Zero-inflated Poisson model predictions (lower left); d) Conditional autoregression model predictions (lower right). (TIF) [file pone.0182903.s001.tif]
