# Supplementary material for: Modeling the spatial distribution of African buffalo (Syncerus caffer) in the Kruger National Park, South Africa
Source: PLoS One. 2017 Sep 13;12(9):e0182903. doi: 10.1371/journal.pone.0182903 (PMC5597095; doi:10.1371/journal.pone.0182903)
Supplement: S1 Table — (DOCX) [file pone.0182903.s002.docx]

**S1 Table.** The comparison of quantitative variables between locations where buffalo were observed in Kruger National Park during August 2012 and January 2013 compared to hourly (non-buffalo) observations and the correlation of these variables with the observed herd sizes.

|  | **Buffalo observed** | | |  |  | |  |
| --- | --- | --- | --- | --- | --- | --- | --- |
|  | **Yes** | **No** |  | | **Correlation with herd size†** | |  |
| **Variable/time** | **Median (IQR)** | **Median (IQR)** | **P value*** | | **Spearman’s rho** | **P value** | |
| August 2012 |  |  |  | |  |  | |
| Latitude | -23.539 (-25.170, -22.973) | -24.091 (-25.105, -23.521) | <0.001 | | 0.258 | <0.001 | |
| Longitude | 31.418 (31.287, 31.581) | 31.451 (31.290, 31.618) | 0.394 | | -0.049 | 0.499 | |
| Temperature (C) | 20.0 (16.0, 23.0) | 21.0 (17.0, 24.0) | 0.264 | | -0.081 | 0.253 | |
| Time | 9:27 (7:38, 11:14) | 10:00 (8:00, 11:00) | 0.355 | | -0.058 | 0.417 | |
|  |  |  |  | |  |  | |
| January 2013 |  |  |  | |  |  | |
| Latitude (dd) | -23.815 (-24.387, -23.504) | -24.020 (-24.868, -23.813) | 0.003 | | 0.214 | 0.012 | |
| Longitude (dd) | 31.434 (31.270, 31.591) | 31.511 (31.365, 31.587) | 0.118 | | -0.115 | 0.179 | |
| Temperature (C) | 23.0 (21.0, 26.5) | 23.0 (21.0, 27.) | 0.739 | | -0.101 | 0.235 | |
| Time | 8:08 (6:59, 9:49) | 9:00 (7:00, 10:30) | 0.134 | | -0.015 | 0.058 | |
|  |  |  |  | |  |  | |
| Combined |  |  |  | |  |  | |
| Latitude | -23.678 (-24.196, -22.998) | -24.026 (-24.996, -23.704) | <0.001 | | 0.250 | <0.001 | |
| Longitude | 31.424 (31.286, 31.578) | 31.489 (31.311, 31.592) | 0.108 | | -0.074 | 0.175 | |
| Temperature (C) | 21.0 (18.5, 25.5) | 22.0 (19.0, 25.0) | 0.508 | | -0.047 | 0.388 | |
| Time | 8:46 (7:19, 10:28) | 9:30 (8:00, 11:00) | 0.036 | | -0.089 | 0.100 | |

Dd = decimal degrees. C = Celcius.

*Based on Mann-Whitney U tests comparing values between sampling points when buffalo were observed versus not observed.

†Nonparametric correlation between the variables and herd size.
