# Supplementary material for: Modeling the spatial distribution of African buffalo (Syncerus caffer) in the Kruger National Park, South Africa
Source: PLoS One. 2017 Sep 13;12(9):e0182903. doi: 10.1371/journal.pone.0182903 (PMC5597095; doi:10.1371/journal.pone.0182903)
Supplement: S2 Table — (DOCX) [file pone.0182903.s003.docx]

**S2 Table.** Univariate logistic regression to identify predictors of observing buffalo herds based on field data collected for 105 detected herds of buffalo in Kruger National Park during August 2012 and January 2013 compared to 234 hourly time points without buffalo observations.

| **Variable** | **level** | **Buffalo observations (n)** | **Total locations**  **(n)** | **Odds ratio**  **(95% CI)** | **Wald P value** |
| --- | --- | --- | --- | --- | --- |
| Season |  |  |  |  |  |
|  | Dry (August) | 56 | 200 | 0.71 (0.45, 1.14) | 0.156 |
|  | Wet (January) | 49 | 139 | Referent |  |
|  |  |  |  |  |  |
| Time |  |  |  |  | 0.123 |
|  | 5:00 – 8:25 AM | 45 | 119 | 1.82 (0.93, 3.59) | 0.081 |
|  | 8:30 – 11:25 AM | 44 | 156 | 1.18 (0.61, 2.29) | 0.628 |
|  | 11:30 – 3:00 PM | 16 | 64 | Referent |  |
|  |  |  |  |  |  |
| Visible water source |  |  |  |  | 0.101 |
|  | River | 18 | 57 | 1.17 (0.63, 2.18) | 0.627 |
|  | Water hole | 8 | 14 | 3.37 (1.13, 10.1) | 0.029 |
|  | Man-made | 9 | 21 | 1.90 (0.77, 4.70) | 0.167 |
|  | None | 70 | 177 | Referent |  |
|  |  |  |  |  |  |
| Vegetation type |  |  |  |  | 0.002 |
|  | Bush | 78 | 203 | 2.38 (1.28, 4.42) | 0.006 |
|  | Mixed | 11 | 59 | 0.87 (0.37, 2.06) | 0.757 |
|  | Tree | 16 | 77 | Referent |  |
|  |  |  |  |  |  |
| Vegetation density |  |  |  |  | 0.014 |
|  | More open | 26 | 54 | 2.53 (1.25, 5.11) | 0.010 |
|  | Middle density | 54 | 192 | 1.06 (0.61, 1.86) | 0.826 |
|  | More dense | 25 | 93 | Referent |  |
|  |  |  |  |  |  |
| Latitude |  |  |  |  | <0.001 |
|  | Northern region | 54 | 115 | 1.72 (0.91, 3.25) | 0.095 |
|  | Central region | 31 | 113 | 4.03 (2.20, 7.39) | <0.001 |
|  | Southern region | 20 | 111 | Referent |  |

CI = confidence interval.
