# Supplementary material for: Modeling the spatial distribution of African buffalo (Syncerus caffer) in the Kruger National Park, South Africa
Source: PLoS One. 2017 Sep 13;12(9):e0182903. doi: 10.1371/journal.pone.0182903 (PMC5597095; doi:10.1371/journal.pone.0182903)
Supplement: S3 Table — (DOCX) [file pone.0182903.s004.docx]

**S3 Table.** Univariate logistic regression to identify predictors of observing bachelor herds in 104 herds* of buffalo in Kruger National Park identified during August 2012 and January 2013.

| **Variable** | **level** | **Bachelor herds observed (n)** | **Total herds (n)** | **Odds ratio**  **(95% CI)** | **Wald P value** |
| --- | --- | --- | --- | --- | --- |
| Season |  |  |  |  |  |
|  | Dry (August) | 21 | 55 | 0.43 (0.19, 0.94) | 0.034 |
|  | Wet (January) | 29 | 49 | Referent |  |
|  |  |  |  |  |  |
| Time |  |  |  |  | 0.249 |
|  | 5:00 – 8:25 AM | 25 | 45 | 2.75 (0.82, 9.22) | 0.101 |
|  | 8:30 – 11:25 AM | 20 | 43 | 1.91 (0.57, 6.45) | 0.295 |
|  | 11:30 – 3:00 PM | 5 | 16 | Referent |  |
|  |  |  |  |  |  |
| Visible water source |  |  |  |  | 0.215 |
|  | River | 6 | 18 | 0.41 (0.14, 1.21) | 0.106 |
|  | Water hole | 2 | 8 | 0.27 (0.05, 1.44) | 0.126 |
|  | Man-made | 4 | 9 | 0.65 (0.16, 2.64) | 0.550 |
|  | None | 38 | 69 | Referent |  |
|  |  |  |  |  |  |
| Vegetation type |  |  |  |  | 0.859 |
|  | Bush | 37 | 77 | 1.19 (0.40, 3.52) | 0.754 |
|  | Mixed | 6 | 11 | 1.54 (0.33, 7.23) | 0.582 |
|  | Tree | 7 | 16 | Referent |  |
|  |  |  |  |  |  |
| Vegetation density |  |  |  |  | 0.026 |
|  | More open | 9 | 25 | 0.22 (0.07, 0.72) | 0.013 |
|  | Middle density | 23 | 54 | 0.29 (0.10, 0.81) | 0.018 |
|  | More dense | 18 | 25 | Referent |  |
|  |  |  |  |  |  |
| Latitude |  |  |  |  | 0.703 |
|  | Northern region | 27 | 54 | 0.90 (0.32, 2.56) | 0.844 |
|  | Central region | 13 | 31 | 0.65 (0.21, 2.05) | 0.462 |
|  | Southern region | 10 | 19 | Referent |  |

*Herd type could not be determined for one herd.

CI = confidence interval.
