# Supplementary material for: Modeling the spatial distribution of African buffalo (Syncerus caffer) in the Kruger National Park, South Africa
Source: PLoS One. 2017 Sep 13;12(9):e0182903. doi: 10.1371/journal.pone.0182903 (PMC5597095; doi:10.1371/journal.pone.0182903)
Supplement: S4 Table — (DOCX) [file pone.0182903.s005.docx]

**S4 Table.** Univariate linear regression for the estimation of effects of predictor variables on observed buffalo herd size* in 104 herds† of buffalo in Kruger National Park identified during August 2012 and January 2013.

| **Variable** | **level** | **Total herds (n)** | **Slope estimate**  **(95% CI)** | **Student’s t**  **P value** |
| --- | --- | --- | --- | --- |
| Bachelor herd | No | 50 | 3.33 (2.95, 3.71) | <0.001 |
|  | Yes | 54 | Referent |  |
|  |  |  |  |  |
| Season |  |  |  |  |
|  | Dry (August) | 56 | 1.12 (0.41, 1.84) | 0.002 |
|  | Wet (January) | 49 | Referent |  |
|  |  |  |  |  |
| Time |  |  |  | 0.342 |
|  | 5:00 – 8:25 AM | 45 | -0.81 (-1.93, 0.29) | 0.147 |
|  | 8:30 – 11:25 AM | 44 | -0.54 (-1.65, 0.58) | 0.342 |
|  | 11:30 – 3:00 PM | 16 | Referent |  |
|  |  |  |  |  |
| Visible water source |  |  |  | 0.159 |
|  | River | 18 | 0.78 (-0.22, 1.78) | 0.123 |
|  | Water hole | 8 | 1.35 (-0.06, 2.76) | 0.061 |
|  | Man-made | 9 | 0.33 (-1.01, 1.67) | 0.626 |
|  | None | 70 | Referent |  |
|  |  |  |  |  |
| Vegetation type |  |  |  | 0.270 |
|  | Bush | 78 | 0.71 (-0.33, 1.78) | 0.179 |
|  | Mixed | 11 | 0.04 (-1.45, 1.53) | 0.959 |
|  | Tree | 16 | Referent |  |
|  |  |  |  |  |
| Vegetation density |  |  |  | <0.001 |
|  | More open | 26 | 2.09 (1.09, 3.08) | <0.001 |
|  | Middle density | 54 | 1.31 (0.45, 2.17) | 0.003 |
|  | More dense | 25 | Referent |  |
|  |  |  |  |  |
| Latitude |  |  |  | 0.356 |
|  | Northern region | 54 | 0.41 (-0.58, 1.41) | 0.413 |
|  | Central region | 31 | 0.79 (-0.30, 1.89) | 0.155 |
|  | Southern region | 20 | Referent |  |

*Analysis performed on the natural logarithm transformed herd size.

†Herd type could not be determined for one herd.

CI = confidence interval.
