# Supplementary material for: Modeling the spatial distribution of African buffalo (Syncerus caffer) in the Kruger National Park, South Africa
Source: PLoS One. 2017 Sep 13;12(9):e0182903. doi: 10.1371/journal.pone.0182903 (PMC5597095; doi:10.1371/journal.pone.0182903)
Supplement: S1 Code — (DOCX) [file pone.0182903.s006.docx]

**S1 Code.** WinBUGS code for CAR model

model {

beta1[1]<-0

beta1[2]<-0

beta1[3]<-0

beta1[4]<-0

beta1[5]<-0

beta1[6]<-0

beta1[7]<-0

beta1[8]<-0

beta1[9]<-0

beta1[10]<-0

beta1[11]<-0

beta1[12]<-0

beta1[13]<-0

alpha1<-0

beta2[1]<-0

beta2[2]<-0

beta2[3]<-0

beta2[4]<-0

beta2[5]<-0

beta2[6]<-0

beta2[7]<-0

beta2[8]<-0

beta2[9]<-0

beta2[10]<-0

beta2[11]<-0

beta2[12]<-0

beta2[13]<-0

K <- 10000 #Constant for implementing zeros trick

for (i in 1:N) {

**#Likelihood**

p[i] <- max(0*.*001*,* min(0*.*999,q[i]))

logit(q[i]) <-alpha0+beta1[1]*dist[i]+beta1[2]*water[i]+beta1[3]*bare[i]+beta1[4]*treec[i]+beta1[5]*herb[i] +

beta1[6]*waterd[i]+beta1[7]*MAR[i]+beta1[8]*metal[i]+beta1[9]*treeq[i]+beta1[10]*herbq[i]+beta1[11]*tree[i]+beta1[12]*mixed[i]+beta1[13]*(dist[i]*water[i])+Phi[1,i]

log(mu[i]) <-alpha1+beta2[1]*dist[i]+beta2[2]*water[i]+beta2[3]*bare[i]+beta2[4]*treec[i]+beta2[5]*herb[i] +

beta2[6]*waterd[i]+beta2[7]*MAR[i]+beta2[8]*metal[i]+beta2[9]*treeq[i]+beta2[10]*herbq[i]+

beta2[11]*tree[i]+beta2[12]*mixed[i]+beta2[13]*(dist[i]*water[i])+Phi[2,i]

z[i] <- step(O[i] - 1) #I(O*>*0)

ll[i] <- (1-z[i])*log(1-p[i]) + z[i]*(log(p[i]) + O[i]*log(mu[i]) - mu[i] - loggam(O[i]+1) - log(1-exp(-mu[i])))

#Log-likelihood

zeros[i] <- 0

zeros[i] ~ dpois(phi[i]) #Zeros trick

phi[i] <- - ll[i]+K

pred[i] <- q[i] * mu[i]

}

**#Priors**

alpha0~dflat()

alpha1~dflat()

beta1[1]~dnorm(0,.1)

beta1[2]~dnorm(0,.1)

beta1[3]~dnorm(0,1)

beta1[4]~dnorm(0,1)

beta1[5]~dnorm(0,1)

beta1[6]~dnorm(0,1)

beta1[7]~dnorm(0,1)

beta1[8]~dnorm(0,.1)

beta1[9]~dnorm(0,.1)

beta1[10]~dnorm(0,.1)

beta1[11]~dnorm(0,.1)

beta1[12]~dnorm(0,.1)

beta1[13]~dnorm(0,.1)

beta2[1]~ dnorm(0,.1)

beta2[2]~ dnorm(0,.1)

beta2[3]~ dnorm(0,1)

beta2[4]~ dnorm(0,1)

beta2[5]~ dnorm(0,1)

beta2[6]~ dnorm(0,1)

beta2[7]~ dnorm(0,1)

beta2[8]~ dnorm(0,.1)

beta2[9]~ dnorm(0,.1)

beta2[10]~ dnorm(0,.1)

beta2[11]~ dnorm(0,.1)

beta2[12]~ dnorm(0,.1)

beta2[13]~ dnorm(0,.1)

**#Bivariate CAR Prior for Phi**

Phi[1, 1:N] ~ car.normal(adj[], weights[], num[], tau)

Phi[2, 1:N] ~ car.normal(adj[], weights[], num[], tau)

for(i in 1:sumNumNeigh) {weights[i] <- 1}

tau ~ dgamma(0.5, 0.0005)

}
